# Self-Assembled Ferritin Nanoparticles for Delivery of Antigens and Development of Vaccines: From Structure and Property to Applications

**DOI:** 10.3390/molecules29174221

**Published:** 2024-09-05

**Authors:** Shinuo Cao, Dongxue Ma, Shengwei Ji, Mo Zhou, Shanyuan Zhu

**Affiliations:** 1 Jiangsu Key Laboratory for High-Tech Research and Development of Veterinary Biopharmaceuticals, Engineering Technology Research Center for Modern Animal Science and Novel Veterinary Pharmaceutic Development, Jiangsu Agri-Animal Husbandry Vocational College, Taizhou 225306, China; shinuo_cao@163.com; 2 Department of Veterinary Medicine, Agriculture College of Yanbian University, Yanji 133000, China; madongxueyjsz@163.com (D.M.); jishengwei0903@hotmail.com (S.J.)

**Keywords:** ferritin, self-assembled protein, nanoparticles, vaccines

## Abstract

Ferritin, an iron storage protein, is ubiquitously distributed across diverse life forms, fulfilling crucial roles encompassing iron retention, conversion, orchestration of cellular iron metabolism, and safeguarding cells against oxidative harm. Noteworthy attributes of ferritin include its innate amenability to facile modification, scalable mass production, as well as exceptional stability and safety. In addition, ferritin boasts unique physicochemical properties, including pH responsiveness, resilience to elevated temperatures, and resistance to a myriad of denaturing agents. Therefore, ferritin serves as the substrate for creating nanomaterials typified by uniform particle dimensions and exceptional biocompatibility. Comprising 24 subunits, each ferritin nanocage demonstrates self-assembly capabilities, culminating in the formation of nanostructures akin to intricate cages. Recent years have witnessed the ascendance of ferritin-based self-assembled nanoparticles, owing to their distinctive physicochemical traits, which confer substantial advantages and wide-ranging applications within the biomedical domain. Ferritin is highly appealing as a carrier for delivering drug molecules and antigen proteins due to its distinctive structural and biochemical properties. This review aims to highlight recent advances in the use of self-assembled ferritin as a novel carrier for antigen delivery and vaccine development, discussing the molecular mechanisms underlying its action, and presenting it as a promising and effective strategy for the future of vaccine development.

## 1. Introduction

Ferritin, the self-assembled protein nanocage, plays a crucial role in iron metabolism and homeostasis for iron detoxification and reservation that prevents oxidative damage to the cells [1,2,3]. Almost all living organisms synthesize this molecule, including archaea, bacteria, algae, eubacteria, plants, and animals [4]. Notably, ferritin genes exhibit a high degree of conservation across species. In vertebrates, all ferritin genes manifest three introns and four exons [5]. As cellular iron repositories, ferritins are hollow, spherical, symmetrical multimeric proteins that sequester excess Fe (II) and synthesize iron biominerals [6]. Ferritin is composed of 24 protein subunits that are arranged in 432 symmetries to form a hollow nanocage characterized by multiple metal–protein interactions [7,8,9]. In vaccine design, ferritin nanoparticles may be well suited to carry and expose immunogens.

**Figure 1:**
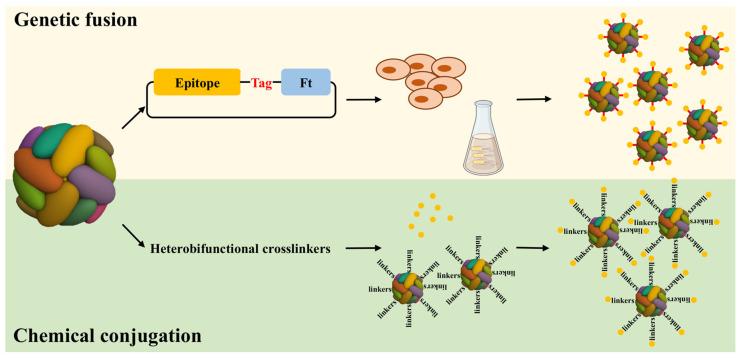
Diagram illustrating how antigens can be individually attached to ferritin subunits through genetic fusion or chemical conjugation. The antigens attached to ferritin subunits through two methods, the genetic fusion and chemical conjugation. In the process of genetic fusion, the gene encoding the antigen epitope was fused to the Ft gene using standard genetic engineering methods. The Ft fusion protein was expressed as an insoluble protein in *Escherichia coli* or mammalian cell overexpression system. During the chemical conjugation process, the Ft was functionalized by the crosslinker at first; furthermore, the antigen was coupled to the crosslinker construct.

Ferritin has been the most widely used as either a bio-platform to synthesize various inorganic and organic nanomaterials or a vehicle for bioactive drugs. In recent studies, the versatility of ferritin has positioned it as a promising platform for vaccine delivery [10,11]. Antigens can be attached individually to the ferritin subunits through genetic or chemical modification (Figure 1). Furthermore, by harnessing the self-assembly capabilities of ferritin, the outer surface can host a multitude of antigens. Notably, the triple symmetry axis inherent to ferritin allows for the presentation of antigens in their multimeric form, typically as trimers. This configuration more faithfully emulates the natural state of multimerized antigens found on viral surfaces compared to monomeric presentations. Concurrently, the L and H subunits of ferritin provide an avenue for the concurrent carriage of distinct antigens, resulting in the simultaneous presentation of two antigens on the surface. By adeptly mixing and assembling recombinant ferritin with various antigenic modifications, it becomes feasible to showcase a diverse array of antigens on the ferritin surface. Moreover, precise control over the antigenic composition of individual ferritin assemblies can be achieved by manipulating the ratio of different ferritin subunits within the assembly system. Due to the unique advantages of ferritin-based vaccines, researchers have developed a range of formulations based on ferritin. Therefore, in this review, we will present the structural analysis, rational vaccine design, and immunogenicity of self-assembling ferritin nanoparticles and elaborate on the underlying molecular mechanisms of the action of ferritin nanoparticles. Additionally, this review will present a promising and effective strategy for future vaccine development.

## 2. The Function and Structure of Ferritin

**Figure 2:**
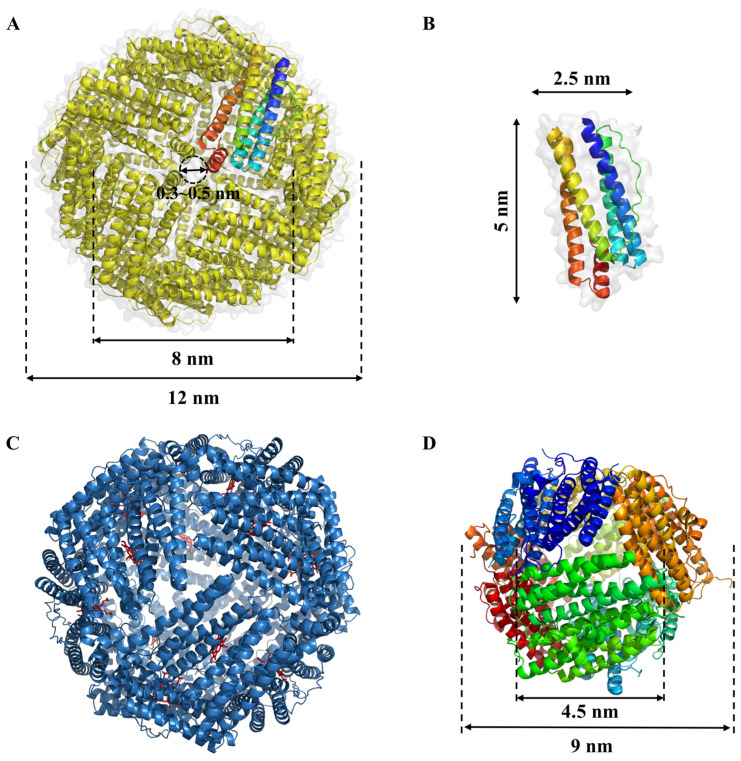
Structural of ferritins family. (**A**) Structure of non-heme-binding ferritins (Ftn) from *E. coli* (PDB ID: 1EUM); (**B**) Subunit of ferritins; (**C**) Structure of heme-binding bacterioferritins (Bfr) from *E. coli* (PDB ID: 1BFR). The bound heme-b co-factor are shown as red sticks between the protein monomers; (**D**) Structure of DNA-binding protein from *E. coli* (PDB ID: 1JTS).

Ferritins are ubiquitously distributed across a diverse spectrum of life forms, where their primary role lies in the meticulous regulation of iron levels by storing and releasing iron at specific loci [12]. Ferritin assumes the pivotal functions of orchestrating iron ion metabolism and upholding iron equilibrium within the organism, concurrently safeguarding cellular constituents. Prokaryotic and eukaryotic domains utilize this multifaceted protein as the principal intracellular iron repository, which preserves iron in a soluble and nontoxic form. Particularly, vertebrate ferritin comprises two or three subunits, designated by their molecular weights: L (“light”), H (“heavy”), and M (“middle”) subunits. Bullfrogs are the only organisms that have the M subunit. Alternatively, ferritin composes only one subunit type in bacteria and archaea. In eukaryotic ferritin, both the H and M subunits adopt the H-type configuration, and all subunits in bacterial and archaeal ferritin have ferroxidase activity [13]. Due to its enzymatic power, ferrous iron and hydrogen peroxide are limited from interacting in a highly destructive reaction known as the Fenton reaction. At the center of every H-type subunit is a diiron binding site responsible for ferroxidase activity [12]. In contrast, ferritin’s light chain lacks ferroxidase capability but facilitates electron transfer across the protein cage [13]. In ferritins, the hollow nanocages are capable of incorporating up to 4500 Fe atoms within their nanocavities and storing them as ferric oxy-hydroxide minerals inside them. According to the distinctions in protein composition and structural attributes, ferritin is categorized into three types: the heme-binding bacterioferritins (Bfr), the non-heme-binding ferritins (Ftn), and the Dps proteins (DNA-binding proteins from starved cells) (Figure 2).

### 2.1. Non-Heme-Binding Ferritins (Ftn)

The classical ferritin, known as Ftn, encompasses a total of 24 subunits, boasting an external diameter of approximately 12 nm and an internal diameter measuring 8 nm (Figure 2A). It assumes an intricately symmetrical, hollow, globular configuration with a relative molecular mass ranging from 450 to 480 kDa. Each of these subunits takes on a cylindrical form, spanning 5 nm in length and 2.5 nm in width, characterized by the presence of 5 to 6 α-helices (Figure 2B) [14]. Natural ferritin is endowed with iron nuclei, primarily composed of 5Fe_2_O_3_·9H_2_O. Within the central cavity of each ferritin molecule, an impressive capacity to harbor roughly 4500 iron atoms is observed. In contrast, ferritin devoid of iron nuclei is referred to as apoferritin [9]. The inner surface of Ftn’s globular structure abounds in acidic residues, notably glutamate and aspartic acid, contributing to an isoelectric point falling within the range of 5.0 to 6.0. This property engenders a pronounced density of negative charges on the inner surface of the ferritin molecule [15]. It has been elucidated that the 24 subunits constituting ferritin form a positively charged octahedral framework. The interconnections between these subunits give rise to a specific array of channels. Each Ftn molecule is endowed with eight 3-fold channels and six 4-fold channels, featuring a pore size spanning from 0.3 to 0.5 nm (Figure 2A). These channels, in conjunction with the interior of ferritin, are characterized by a net negative charge. This distinctive attribute facilitates the diffusion of metal ions and positively charged small organic molecules into the interior cavity [16,17].

### 2.2. Heme-Binding Bacterioferritins (Bfr)

Heme-binding bacterioferritins (Bfr) differ from Ftn by incorporating twelve b-type heme groups between subunits in a twofold symmetric binding site, along with classical ferritin (Figure 2C) [18,19]. Distinguishing Bfr from classical ferritin Ftn is the presence of a heme group bridging every pair of subunits in the former, whereas such heme linkages are notably absent among Ftn subunits [20]. Bfr fulfills essential roles, encompassing iron storage, active involvement in safeguarding cells against redox stress, and the maintenance of cellular homeostasis. Notably, its iron storage function predominantly hinges on its capacity to bind to heme moieties [18]. Heme plays a crucial role in the rapid iron release from bacterial ferritin by acting as a facilitator for the reduction of the Fe (III) ions stored within the ferritin core to Fe (II). This reduction is a key step that allows the iron to be mobilized and utilized by the cell. Heme’s involvement in this process is significant because it provides a mechanism for the controlled release of iron, which is essential for various cellular processes, including respiration and DNA synthesis [21].

### 2.3. DNA-Binding Proteins from Starved Cells (Dps)

DNA-binding proteins from starved cells (Dps), a mini-ferritin, was discovered in *Escherichia coli* during the stationary phase and is found in a wide variety of bacteria, archaea, and antioxidant enzymes. Dps plays a key role in protecting bacterial chromosomes from oxidative damage [21,22,23,24,25]. Dps exhibits a tetrahedrally symmetric shell configuration comprising precisely 12 identical subunits. Its external dimensions measure approximately 9 nm in outer diameter, encompassing an internal cavity with a diameter of 4.5 nm (Figure 2D) [26]. Given its relatively compact size, Dps can accommodate a modest inventory of iron atoms within its central chamber. Beyond iron storage, a pivotal role of Dps lies in safeguarding DNA against iron-triggered oxidative harm, thereby mitigating the deleterious Fenton reaction [27]. In *E. coli*, Dps is upregulated during nutrient scarcity, forming a dodecameric structure that shields DNA from reactive oxygen species (ROSs) and other harmful agents, which is crucial for long-term stress survival. *Listeria monocytogenes*, the pathogen causing listeriosis, uses Dps to protect against oxidative damage and to persist in food and harsh gastrointestinal conditions. Similarly, *Mycobacterium smegmatis*, a relative of *Mycobacterium tuberculosis*, expresses Dps for protection during oxidative stress, suggesting how pathogenic mycobacteria might endure the human immune response. *Helicobacter pylori*, linked to gastric ulcers and cancer, employs Dps-like proteins to protect its DNA from oxidative damage in the acidic stomach environment, aiding its persistent colonization. Other bacteria, including *Bacillus anthracis* (anthrax), *Streptococcus mutans* (dental caries), and *Salmonella typhimurium*, also utilize Dps for protection. In *B. anthracis*, Dps is involved in spore DNA protection; in *S. mutans*, it combats oxidative stress in the oral cavity; and in *S. typhimurium*, it helps resist oxidative stress within macrophages during infection [26,28,29].

The ubiquitous presence and conservation of Dps proteins across diverse bacteria underscore their essential role in DNA protection, stress response, and gene regulation, highlighting their multifaceted importance in bacterial physiology and survival.

## 3. Self-Assembled Ferritin Nanoparticles for Vaccine Development

A fundamental attribute inherent to ferritins lies in their ability to undergo self-assembly, forming the characteristic 24-mer shell both in vitro and in vivo, thereby precluding the existence of unbound subunits in the solution. Furthermore, investigations have revealed the propensity of ferritins derived from various sources, including mammals, fish [30], insects [31], and plants [32,33,34], to give rise to heteropolymers comprising two or more distinct subunit types.

### 3.1. Self-Assembly and Hetero-Polymerization of Ferritin

Ferritin secreted from insects has an architectural configuration consisting of 12 heterodimers of H- and L-chains arranged in a tetrahedral pattern. Vertebrate ferritins, on the other hand, are characterized by one type of subunit arranged in octahedral symmetry [31]. Notably, this structural divergence elucidates the imperative need for the expression of both H- and L-chain subtypes to confer functional ferritins in insects [35]. In mammalian cells, the assembly of H- and L-chains occurs in proportion to their relative expression levels. However, it is noteworthy that exogenous ferritin subunits expressed during transient transfection in COS7 cells do not co-assemble with their endogenous counterparts [36], a phenomenon contrary to those expressed after stable transfection [37]. This discrepancy implies a temporal dependency in the in vivo formation of heteropolymers, attributed to the sluggish turnover of endogenous ferritins impeding association with rapidly synthesized ferritin chains. In vitro, ferritin assembly is a spontaneous process with a discernible preference for the formation of heteropolymers over homopolymers when both H- and L-subunits are present. This proclivity facilitates the production of heteropolymers with the desired H/L proportion [38,39]. Researchers designed a copper-dependent subunit dimer interaction experiment and found that in the absence of copper, the subunits folded into inactive monomers [40,41]. These results suggest that subunit dimers might serve as the initial intermediates in the self-assembly pathway. Understanding the self-assembly mechanism of supramolecular structures is challenging. X-ray diffraction studies on horse spleen apoferritin indicate that stable dimers are the initial intermediates, interacting along most of their length with a larger contact area than those near the 3- or 4-fold axes. The next step likely involves the formation of hexamers, where dimers aggregate around the triagonal axis, which has a larger contact region compared to the 4-fold axis. Ferritin nanoparticles have three modifiable interfaces: the interior surface, the exterior surface, and the regions between subunits. These can be altered through chemical and genetic engineering to create functional nanocages for various biotechnological uses. Research on modifying the inter-subunit regions has focused on controlling nanoparticle assembly and disassembly. Typically, ferritin subunits assemble spontaneously, and disassembly can be induced under extreme pH conditions (pH < 2 or pH > 10) or with reducing agents, with reassembly occurring at neutral pH [11]. Recently, scientists have utilized the reversible pH-controlled self-assembly properties of apoferritin cages to encapsulate and deliver bioactive nutrients or anticancer drugs. The ferritin cages can protect their cargo from external conditions and provide a controlled microenvironment. Moreover, after encapsulation, the ferritin shells significantly improve the water solubility, thermal stability, photostability, and cellular uptake activity of these small bioactive compounds.

### 3.2. Engineered Ferritin-Based Vaccine

**Figure 3:**
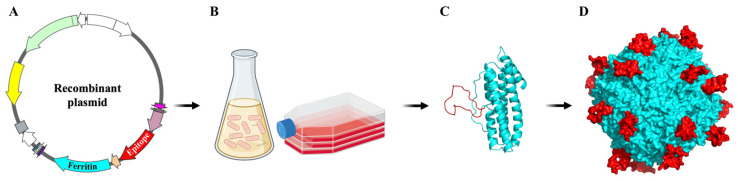
Schematic representation of ferritin-based self-assembling nanoparticles. (**A**) Recombinant plasmid of ferritin-based vaccine. The gene of ferritin was shown in light blue color and the antigen gene was shown in red color. (**B**) The recombinant plasmid was expressed with the common gene expression system. (**C**,**D**) Ferritin subunits spontaneously assemble into spherical structures, and additional antigens are expressed and integrated on the outer surface of ferritin during ferritin assembly.

The biogenetic modification of ferritin does not impede the inherent self-assembly among ferritin subunits, and all 24 subunits can be genetically altered. Therefore, ferritin nanoparticles have significant potential as a robust platform for vaccine development and antigen delivery. The genetic engineering of ferritin itself can be readily achieved. This entails the direct attachment of the antigenic subunit sequence to the terminus of the ferritin gene sequence, followed by the efficient production of target proteins in substantial quantities by introducing a recombinant plasmid into an expression system, such as a bacterial or cellular platform. During the expression process, ferritin subunits spontaneously assemble into spherical structures, while the appended antigenic subunits are concurrently expressed and integrated within the ferritin assembly [42,43]. Upon completion of this assembly, the antigenic subunits are prominently displayed on the outer surface of ferritin (Figure 3). This genetic engineering approach is straightforward and user-friendly, with the distinct advantage of not disrupting the intrinsic self-assembly and physiological functionality of ferritin through strategic design. In contrast to alternative techniques involving expression, purification, and subsequent modification and ligation, genetic engineering offers a direct means of presenting the target peptide on the outer surface of ferritin. Notably, the three-fold axis channels inherent to apo-ferritin cages, which are characterized by their hollow structure, are well suited for conjugating with antigens requiring trimeric structures to induce robust immune responses. Ensuring that antigens are displayed on the exterior surface of the ferritin nanoparticle rather than sequestered within the internal cavity is critical for eliciting an effective immune response. One common strategy is to genetically fuse the antigen of interest to the N- or C-terminal regions of the ferritin subunit, which are naturally oriented towards the exterior when the ferritin assembles into its 24-mer nanocage structure. By carefully selecting the terminal region for fusion, researchers can take advantage of the spatial orientation of these terminal residues to ensure that the fused antigens are presented outwardly. Additionally, linker sequences can be incorporated between the ferritin subunit and the antigen to provide flexibility and prevent steric hindrance, which might otherwise cause the antigen to fold back into the nanoparticle’s internal cavity. Computational modeling and structural analysis can further guide the design, ensuring the correct orientation and optimal exposure of the antigen on the nanoparticle surface [44].

Engineered ferritin nanoparticles have emerged as a promising platform for vaccine development due to their ability to present antigens in a highly organized manner, mimicking the repetitive and dense structures of viruses. One key challenge in developing ferritin-based vaccines is the potential impact of inserting antigenic sequences on the protein’s natural folding and self-assembly. Ferritin naturally forms a highly stable 24-mer nanocage structure, but incorporating foreign epitopes could disrupt this stability, leading to improper folding, loss of assembly, or decreased immunogenicity. Researchers have been investigating strategies to mitigate these issues by optimizing the placement and sequence of antigens to maintain the structural integrity of the ferritin cage. Successful examples of engineered ferritin vaccines include the expression of influenza hemagglutinin (HA) on the surface of ferritin nanoparticles. These HA-ferritin vaccines have shown robust immune responses and protection in animal models, demonstrating the potential of ferritin as a vaccine platform. Additionally, ferritin nanoparticles displaying the receptor-binding domain (RBD) of the SARS-CoV-2 spike protein have been successfully expressed and are being evaluated for their efficacy against COVID-19, showing promising results in preclinical studies [44,45].

### 3.3. Production of Ferritin-Based Vaccine

Ferritin vaccines represent a category of genetically engineered subunit recombinant vaccines meticulously crafted by immobilizing antigenic subunits onto ferritin, serving as a versatile carrier. Ferritin with genetically fused antigens, intended for vaccine or targeting purposes, has primarily been synthesized in *E. coli* and HEK293. Bacterial cells lack the capability to express glycosylated proteins and complex antigens with proper folding, necessitating the production of ferritin fusion constructs in animal cells. Chimeric ferritin is a mix of glycosylated antigens (e.g., HA, gp140, S protein) fused to ferritin produced in mammalian cells. Knowledge of the optimal conditions for chimeric ferritin production may also influence the selection of the host. Notably, utilizing CHO cells for ferritin-based vaccine production has been spearheaded by a particular research group [46,47,48,49]. Ferritin nanoparticles can be produced with high purity using insect cells and the baculovirus expression vector system, providing an alternative platform for ferritin-based vaccine production [50]. Chen et al. developed a ferritin-based vaccine candidate against foot-and-mouth disease virus using Sf-9 cells; the vaccine yielded partial protection in mice compared to the commercial inactivated vaccine [51]. Additionally, to improve the solubility of the final nanoparticles, the same research group used the signal peptide of the human CD5 leader sequence to guide the release of E2 glycoprotein-ferritin nanoparticles. Similar to the subunit vaccine, this vaccine candidate was expressed in Sf-9 cells, and the production of neutralizing antibodies in immunized rabbits was significantly advanced [52].

During the process of producing a ferritin-based vaccine, adding antigens to the terminal regions of ferritin can sometimes lead to solubility issues because the added antigen sequences may introduce hydrophobic patches or alter the overall charge distribution on the ferritin surface, leading to unfavorable interactions and aggregation. To address these solubility issues, several strategies can be employed. One approach is the use of flexible linker sequences between the ferritin subunit and the antigen. These linkers can provide the necessary spatial separation, reducing steric hindrance and unfavorable interactions that might lead to aggregation. Additionally, optimizing the length and composition of these linker sequences is crucial, as overly short or highly charged linkers may not effectively mitigate solubility issues. Another strategy is to engineer the antigen itself by modifying its sequence to enhance solubility without compromising its immunogenicity. This could involve the introduction of polar residues or the reduction of hydrophobic regions. Furthermore, expressing and purifying the engineered ferritin–antigen fusion proteins in the presence of solubility-enhancing agents, such as certain buffer systems or co-expressed chaperones, can help maintain solubility and proper folding [9,53].

## 4. Self-Assembled Ferritin Nanoparticles Display Different Types of Antigens

The use of nanoparticles to present viral antigens in multivalent arrays has become a promising technology for vaccine development. By displaying highly ordered, repetitive arrays of antigens on the nanoparticle surface, we can mimic the geometric arrangement found on virion surfaces, leading to stronger humoral responses compared to soluble viral antigens. Recently, various antigens have also been displayed on self-assembling protein nanoparticles, resulting in the induction of protective antibodies and effective T-helper responses. This further validates the nanoparticle platform as a universal strategy for eliciting potent immunogenicity.

### 4.1. The Short Peptide Antigen Presented by Ferritin Nanoparticles

Ferritin-displayed short peptide antigens represent the most straightforward iteration of ferritin-based vaccines. Certain short peptide antigens, consisting of sequences of fewer than 200 amino acids, can be directly affixed to ferritin subunits through genetic engineering. These peptides are subsequently exhibited on the external surface of ferritin through the inherent self-assembly mechanism of ferritin, resulting in a multivalent presentation of antigens. Due to the diminutive size of short peptide antigens, they exert minimal influence on the spatial resistance encountered during ferritin self-assembly. When devising peptide sequences, an appropriate sequence can be readily identified through relatively simple experimentation. A research group, as demonstrated by Wang et al. in 2019, truncated the antigenic epitope of the human enterovirus (EV71) into short peptides of varying lengths, each comprising fewer than 20 amino acids. Subsequently, they employed genetic engineering techniques to affix these peptides to various regions of ferritin, including the N-terminal, C-terminal, and loop regions. The research team then systematically probed the impacts of both the recombination position and the length of the truncated epitope on the ensuing immune response. The findings demonstrated that when antigenic peptides were affixed to the N-terminus and the loop region, they presented 24 short peptide antigens on the external facade of ferritin, whereas peptides attached to the C-terminus were confined to the inner cavity of ferritin [54]. Remarkably, the ferritin vaccine featuring antigens displayed on its outer surface elicited a markedly heightened immune response compared to its counterpart with antigens exhibited on the internal surface. The ferritin vaccine integrating antigenic peptides at the loop region conferred the most robust protection in murine subjects.

### 4.2. The Elongated Peptide Antigens Presented by Ferritin Nanoparticles

While it is relatively straightforward to produce substantial quantities of recombinant ferritin vaccines through direct modification of short peptides onto ferritin subunits, research findings indicate a negative correlation between peptide length and antibody potency. Specifically, shorter peptides tend to result in diminished antibody potency and suboptimal immune responses. This phenomenon arises from the inherent limitation of short peptide antigens in capturing and faithfully representing the genuine conformation of antigens present on the virus surface. Moreover, many antigens on virus surfaces exist as multimers, necessitating the development of vaccines that faithfully mimic both the length and spatial arrangement of antigenic peptide fragments that can assemble into polymeric structures. The triple or quadruple symmetry axes inherent to ferritin offer an ideal platform for spatially presenting antigenic peptides in trimeric or tetrameric configurations, respectively. Recognizing that a majority of antigens adopt trimeric forms, researchers have enhanced the ferritin vaccine, successfully achieving the display of elongated peptide antigens as trimers on the external surface of ferritin [55,56]. This strategic modification aligns with the imperative to faithfully replicate the native multimeric structure of viral antigens and holds significant promise for vaccine development.

### 4.3. Enhancing Expression and Assembly of Ferritin Vaccines Harboring Extended Peptide Antigens in Mammalian Cells

In the pursuit of crafting robust recombinant ferritin vaccines harboring extended peptide antigens, extensive efforts have been devoted to investigating and enhancing the methodologies for linking such antigens with ferritin and optimizing their expression and purification. In 2013, a breakthrough was achieved by American scientists who developed a technique for the expression and purification of ferritin within animal cells. This breakthrough effectively mitigated the interference posed by lengthy peptides on ferritin assembly. Their approach entailed genetic engineering, whereby an influenza virus hemagglutinin spike was directly affixed to the terminal end of ferritin. Subsequently, the recombinant plasmid was introduced into mammalian cells for expression. Within the confines of mammalian cells, ferritin, adorned with the modified long peptide antigens, was successfully expressed and autonomously assembled [44]. Detailed examination via electron microscopy revealed that the hemagglutinin spikes presented themselves as trimers on the ferritin surface. This precise spatial configuration facilitated the ferritin vaccine in eliciting antibody titers exceeding those generated by an inactivated influenza virus vaccine by more than tenfold.

Ferritin, when coupled with extended peptides, remains unexpressed within *E. coli*. However, it exhibits the capability for expression and standard assembly within mammalian cells, which is likely attributed to the abundant endomembrane system characteristic of mammalian cellular environments. These mammalian cells house essential organelles, notably the endoplasmic reticulum and the Golgi apparatus, both of which house heat stress proteins crucial for aiding in the proper folding of peptides that may otherwise misfold. The presence of these heat-stress proteins facilitates the appropriate assembly of recombinant ferritin. In theory, the expression of a ferritin-based vaccine within mammalian cells holds the potential to elicit a more potent immune response. This is owing to the fact that proteins expressed within mammalian cells undergo glycosylation within the endomembrane system, resulting in an antigen that closely mimics the authentic viral state following infection.

### 4.4. The Bivalent Antigens Presented by Ferritin Nanoparticles

In prokaryotic organisms, ferritin typically comprises a solitary subunit, whereas in higher eukaryotic organisms, ferritin can encompass both L- and H-chain subunits. When distinct antigens are affixed to the termini of these L- and H-chains, they collectively form complete spherical structures. Each of these L- and H-chains independently adopts a triple axis of symmetry configuration, thereby presenting two trimeric antigens concurrently. This engineered ferritin consists of 12 L- and 12 H-chains, to which the H1N1 and B antigens can be attached, specifically to the termini of the L- and H-chains, respectively. Through the inherent self-assembly mechanism of ferritin, the final assembled ferritin structure boasts bivalent antigens. Remarkably, these two antigens have the ability to organize into four trimeric antigen clusters, all centered around the triple symmetry axis of the ferritin. Moreover, this innovative approach successfully demonstrated the concurrent presentation of two human immunodeficiency virus (HIV) antigens and HIV antigen-linked influenza antigens [57]. This method relies on a precise ratio of heavy chain to light chain assembly, thereby furnishing a platform for the coordinated display of multiple trimeric antigens. Furthermore, the H- and L-chains of ferritin can be recombined with diverse antigenic genes, such as the HA subunit of different influenza virus strains and the envelope protein (Env) of HIV. This facilitates the creation of recombinant ferritin nanoparticles harboring two dissimilar antigens. Importantly, these recombinant nanoparticles can stimulate the production of neutralizing antibodies against the corresponding viruses in mice, as demonstrated by Georgiev et al. in 2018. This pioneering work provides a foundational framework for the potential development of multifaceted ferritin nanovaccines in the future [58].

## 5. Molecular Mechanism of Self-Assembling Ferritin Nanoparticles to Enhance Immune Response

**Figure 4:**
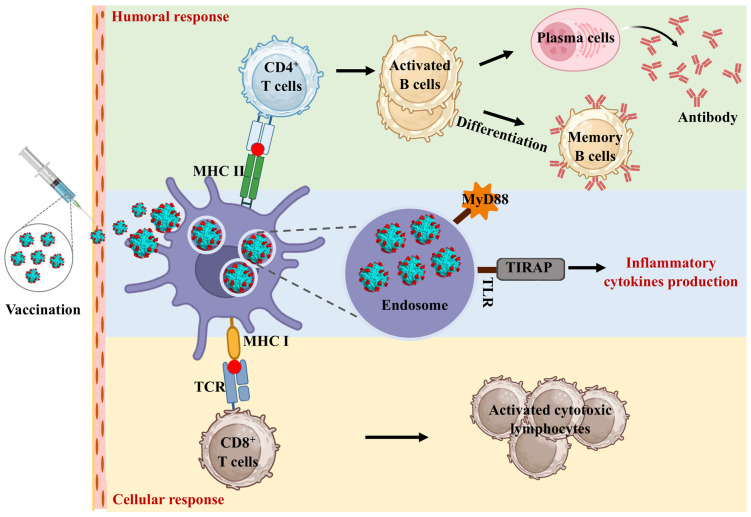
The molecular mechanism of self-assembling ferritin nanoparticles to enhance immune response. The nanoscale size and structural uniformity of ferritin nanoparticles facilitate their uptake by antigen-presenting cells (APCs). Once internalized by these cells, ferritin nanoparticles are processed in endosomal compartments, where antigens are degraded into peptides. These peptides are then loaded onto major histocompatibility complex (MHC) class II molecules, which are essential for presenting antigens to CD4^+^ T cells, leading to T cell activation and a subsequent adaptive immune response. In addition to MHC class II presentation, ferritin nanoparticles can enhance cross-presentation pathways, where exogenous antigens are presented via MHC class I molecules. This is crucial for eliciting cytotoxic CD8^+^ T cells responses, which are essential for targeting intracellular pathogens. The structure of ferritin allows for efficient entry into the cross-presentation pathway, thereby enhancing the cytotoxic T lymphocyte (CTL) responses.

The study of ferritin nanoparticles in enhancing the immune response has garnered significant attention due to its potential applications in vaccine development. Ferritin nanoparticles can enhance the presentation efficiency of dendritic cells (DCs), promote their maturation, and extend the duration of immunity. Additionally, ferritin nanoparticles can activate inflammatory responses, boosting both cellular and humoral immunity levels (Figure 4).

### 5.1. Ferritin Nanoparticles Activated the Inflammatory Response

Growing evidence not only associates elevated circulating ferritin levels with an acute-phase response but also posits its pivotal involvement in the inflammatory cascade [59]. It was found that ferritin regulates NF-kB activation and the expression of proinflammatory molecules as a factor independent of iron [60]. The deletion of the ferritin H-chain has demonstrated a mitigating effect on the inflammatory burden in a sepsis model, leading to reductions in interferon-γ (IFN-γ), interleukin-6 (IL-6), IL-1β, and IL-12 [61]. Additionally, ferritin induces expression of Toll-like receptor 9 (TLR9) and other TLRs in leukemic cells [62,63,64]. The effects of ferritin on systemic inflammation in mice are similar to hyperferritinemia’s clinical manifestations of cytokine storm and liver damage [65]. This highlights the complex role of ferritin in regulating inflammatory responses and lays the foundation for further exploration of its role in immunomodulation. Furthermore, Han et al. used ferritin protein cage nanoparticles (FPCNs) as antigen delivery platforms for DC-based vaccine development, focusing on DC-mediated immune responses. Antigenic peptides OT-1 (SIINFEKL) and OT-2 (ISQAVHAAHAEINEAGR) from ovalbumin were genetically introduced into or onto FPCNs. These FPCNs delivered antigenic peptides to DCs, processed within endosomes, inducing specific CD8^+^ and CD4^+^ T cell proliferations in vitro and in vivo. Mice immunized with OT-1-FPCN showed efficient differentiation of OT-1-specific CD8^+^ T cells into cytotoxic T cells, selectively killing target cells. OT-2-FPCN induced functional Th1 and Th2 CD4^+^ T cells, which was confirmed by the production of cytokine production (IFN-γ/IL-2 and IL-10/IL-13) [10,65].

### 5.2. Ferritin Nanoparticles Enhance Dendritic Cell Uptake

Dendritic cells are crucial for antigen presentation and are recognized as the primary professional antigen-presenting cells (APCs) in vivo. They are essential for inducing and regulating antigen-specific T and B cells. DCs capture antigens, process them, and present the antigenic peptides to T cells through major histocompatibility complex (MHC) class I and class II molecules. Human ferritin possesses the capacity to identify and bind with transferrin receptor 1 (TfR1), a receptor expressing on the surface of DCs. Researchers have harnessed this characteristic to precisely direct antigens to DCs, thereby augmenting immune system activation through vaccination [66,67]. Substantial evidence indicates that antigens anchored to nanoparticles exhibit greater efficacy compared to their monomeric, soluble counterparts [68]. An antigen that is anchored to the surface of a nanoparticle may be taken up more efficiently by the cell as a result of the affinity that is established between the surface and the cell membrane [69]. Since the nanoparticles align with the natural targets of DCs, viruses and bacteria, they are considered optimal for cellular uptake by DCs [69,70].

### 5.3. Ferritin Nanoparticles Increase the Duration of Antigen Presentation

Additionally, the ordered distribution of antigens on ferritin nanoparticle surfaces facilitates multivalent binding of B cell receptors, thereby enhancing interactions with cognate B cells [71]. A nanoparticle-carried antigen differs from a monomeric, soluble antigen in terms of its pharmacokinetics. In contrast to antigen clusters, which diffuse into body fluids and undergo dilution upon administration, nanoparticles enable the maintenance of antigen clusters. Nanoparticles smaller than 20 nm have a longer time to circulate [72]. Furthermore, nanoparticles can enhance the transport of antigens to LNs, increase antigen uptake by LN-resident DCs, and stimulate immune responses by T and B cells [69]. Moreover, nanoparticle-based vaccines have the advantage of being retained at the injection site, enhancing DC uptake and presentation of antigens [68].

### 5.4. Ferritin Nanoparticles Target Macrophages and Improve the Immune Response

Ferritin nanoparticles can enter macrophages through various endocytic pathways, including clathrin-mediated endocytosis, caveolin-mediated endocytosis, and macropinocytosis. Once inside the macrophages, ferritin nanoparticles can be processed in the endosomes and lysosomes, leading to the presentation of antigens via MHC class II molecules, thereby activating T cells and stimulating an adaptive immune response [73,74]. To enhance the immune response elicited by engineered ferritin vaccines, several strategies can be employed. One approach is to modify the surface of ferritin nanoparticles to target specific receptors on macrophages, such as the mannose receptor or scavenger receptors, which can enhance uptake and improve antigen presentation efficiency. Additionally, incorporating molecular adjuvants into the ferritin structure, such as TLR agonists, can further boost the immune response by stimulating innate immunity pathways. Another strategy to enhance the immunogenicity of ferritin-based vaccines is to optimize the antigen presentation. This can be achieved by engineering the antigen to include epitopes that are more efficiently recognized and processed by APCs. Furthermore, co-delivery of ferritin nanoparticles with cytokines can help recruit and activate macrophages and dendritic cells, thereby enhancing the overall immune response. Lastly, modifying the size and surface charge of ferritin nanoparticles can also influence their interaction with immune cells and the subsequent immune response. Smaller nanoparticles with appropriate surface modifications can enhance cellular uptake and antigen presentation, leading to a stronger and more durable immune response [75,76,77].

## 6. The Advancement of Self-Assembled Ferritin Nanoparticles Vaccines Development

Displaying viral antigens on nanoparticles in multivalent arrays has become a significant advancement in vaccine technology. These highly ordered, repetitive antigen arrays on nanoparticle surfaces can mimic the geometric arrangement found on virion surfaces, thereby eliciting stronger humoral responses compared to soluble viral antigens. Recently, bacterial antigens have also been presented on self-assembling protein nanoparticles, resulting in the generation of protective antibodies and effective T-helper cell responses (Table 1). This further substantiates the nanoparticle platform as a versatile approach for inducing robust immunogenicity.

### 6.1. Self-Assembled Ferritin Nanoparticles Vaccines against Virus Infection

The influenza virus poses a significant public health threat due to frequent RNA variations. Conserved epitope-targeting vaccines exist, but more efficient strategies like nanoparticle-based vaccines are needed. Wang et al. used regulated lysis *Salmonella* as an oral vector to deliver M2e-ferritin nanoparticles and evaluated immune responses [79]. Sequential immunization with *Salmonella*-delivered nanoparticles followed by an intranasal boost with purified nanoparticles enhanced immunity. This approach significantly activated lung CD11b DCs, increased effector memory T cells and tissue-resident memory T cells in the lungs, and boosted mucosal IgG and IgA antibody production. Furthermore, seasonal influenza vaccines typically provide strain-specific protection and are reformulated annually, which is a complex and time-consuming process. Multiepitope vaccines, combining multiple conserved antigenic epitopes, can provide robust and diverse immune responses but face issues of low immunogenicity and short-term effectiveness. Nie et al. developed an influenza multiepitope nanovaccine (MHF) by combining linear epitopes from hemagglutinin and matrix protein 2 with *H. pylori* ferritin [57]. MHF self-assembles into uniform nanoparticles, and in mice, it induces cross-reactive neutralizing antibodies, cellular immunity, and long-lasting memory B cell responses, offering protection against H3N2 and partial protection against H1N1. This approach promises scalable manufacturing and effective, long-lasting immunity. Duck Tembusu virus (DTMUV) reduces egg production and causes neurological issues in ducklings. Qu et al. developed self-assembled nanoparticles using DTMUV E protein domain III and ferritin [80]. The nanoparticles induced higher antibody titers, lymphocyte proliferation, IL-4, and IFN-γ levels. These nanoparticle-vaccinated ducks had milder symptoms, higher survival rates, and lower DTMUV RNA levels post-DTMUV challenge.

African swine fever (ASF) threatens the global swine industry. Developing effective vaccines is crucial. Song et al. created NanoFVax, a self-assembled nano-ASFV vaccine targeting DCs. This vaccine couples 24-mer ferritin with dominant ASFV epitopes (p72, CD2v, pB602L, and p30) and the chemokine receptor XCL1 via the SpyTag/SpyCatcher system [90]. NanoFVax induces a robust T cell response and high-level antibodies lasting over 231 days. This nanoparticle is showing promise as an effective ASFV vaccine candidate. Classical swine fever (CSF) causes significant economic losses in the swine industry. The CSFV E2 protein is a major protective antigen [52]. Zhong et al. developed a stable CHO cell line to express E2 protein and delivered it using ferritin nanoparticles [91]. In pigs, nanoparticle-delivered E2 protein elicited higher neutralizing antibody titers and significantly induced CSFV-specific IFN-γ-secreting cells compared to monomeric E2 protein. All pigs inoculated with E2-ferritin nanoparticles were fully protected from a lethal CSFV challenge, demonstrating robust humoral and cellular immune responses. In addition, ferritin nanoparticles are attractive candidate vaccine platforms for developing vaccines against other swine diseases, such as foot-and-mouth disease (FMD), porcine reproductive and respiratory syndrome virus (PRRSV), and porcine epidemic diarrhea virus (PEDV) [42,88,89].

Peste des petits ruminants (PPR) is a highly pathogenic disease affecting goats and sheep. Li et al. introduce a nanoparticle vaccine using *H. pylori* ferritin as a delivery vector for the PPR virus (PPRV) hemagglutinin (H) protein [81]. These nanoparticles exhibited higher expression levels and enhanced immunogenicity and protective responses compared to the H protein alone, indicating that ferritin nanoparticles can improve antigen expression and immune efficacy. Bovine parainfluenza virus type 3 (BPIV3) is a major respiratory pathogen in cattle, with no specific therapies available. Qu et al. developed a BPIV3 nanoparticle vaccine by fusing the ectodomain of BPIV3 hemagglutinin-neuraminidase to ferritin using a baculovirus system [82]. BPIV3 nanoparticles induced DC maturation, upregulated surface molecules, increased inflammatory cytokine secretion, and enhanced T cell activation, induced higher titers of specific antibodies, and provided better protection against BPIV3 in mice. Canine distemper virus (CDV) is highly infectious, affecting domestic and wild animals globally. Current CDV vaccines are attenuated and face challenges like complex preparation, high cost, and safety risks. Wang et al. developed ferritin-coupled nanoparticles (YaH3F, YaH4F, and YaH5F) and a full-length HA-ferritin DNA vaccine (YaHF) using CDV hemagglutinin sequences [83]. All proteins self-assembled into nanoparticles. Vaccination induced strong, long-lasting antibody responses and anti-CDV neutralizing activity, particularly in the YaH4F and YaHF groups, which also enhanced ADCC effects and induced Th1 and Th2 responses.

Despite new antivirals, hepatitis C virus (HCV) still poses a public health challenge. An effective preventive vaccine is needed. A subunit vaccine using recombinant soluble E2 (sE2) induces broadly neutralizing antibodies. To enhance immunogenicity, sE2-ferritin fusion protein in Drosophila S2 cells was created [84]. This nanoparticle displayed sE2 with natural conformation and higher affinity for antibodies and receptors. Mouse studies showed sE2-ferritin induced stronger anti-HCV antibodies than sE2. Rotavirus causes severe diarrhea in young children. The VP6 protein is a potential vaccine candidate for cross-protective immunity. Using ferritin nanoparticles as an antigen scaffold enhances subunit vaccine immunogenicity and protection. Li et al. developed a self-assembling recombinant rotavirus VP6-ferritin (rVP6-ferritin) nanoparticle vaccine expressed in *E. coli* [85]. Oral administration in mice induced strong humoral and mucosal immunity. Transgenic expression in mouse milk also induced strong immunogenicity in pups, reducing diarrhea symptoms and growth. The COVID-19 pandemic, caused by SARS-CoV-2, underscores the need for efficient broad-spectrum vaccines. Wang et al. developed a universal strategy using omicron BA.5 RBD-conjugated ferritin nanoparticles. These nanoparticles, created by bonding RBD-Fc to protein A-ferritin, showed high uniformity and stability [80]. The vaccine stimulated strong innate and adaptive immune responses, inducing cross-neutralizing antibodies and protecting golden hamsters from multiple SARS-CoV-2 variants.

### 6.2. Self-Assembled Ferritin Nanoparticles Vaccines against Bacterial Infections

Recent studies show that bacterial antigens on self-assembling protein nanoparticles elicit strong antibody and T-helper responses, supporting this platform for potent immunogenicity. Veggi et al. designed, analyzed, and tested ferritin nanoparticles displaying Neisseria meningitidis trimeric adhesin NadA. Two constructs, NadA head only and head with stalk, were fused to ferritin and expressed in *E. coli*, forming expected nanoparticles [86]. In mice, both nanoparticles induced NadA antibody levels 10- to 100-fold higher than NadA trimer subunits and potent complement-mediated bactericidal activity.

Serious *Pseudomonas aeruginosa* (PA) infections and rising multidrug resistance demand effective vaccines. No approved vaccine exists, partly due to inefficient delivery systems. Self-assembled ferritin nanoparticles enhance immune response activation. Li et al. developed rePO-FN, a nanovaccine combining PcrV and OprI antigens with ferritin. Adjuvant-free rePO-FN induced rapid, effective immunity and protected mice against PA pneumonia, outperforming aluminum-adjuvanted PcrV-OprI [92]. Intranasal immunization enhanced mucosal immunity. rePO-FN showed good biocompatibility and safety, highlighting its promise as a vaccine candidate and supporting ferritin-based nanovaccines’ efficacy. In addition, flagellin is a key vaccine target for PA, but flagellin-based vaccines have failed due to poor adjuvants or delivery systems. Wei et al. fused A-type flagellin (FliC) to ferritin to create reFliC-ferritin (reFliC-FN) nanoparticles, inducing a strong Th1 immune response and enhancing protection against PA strains with A-type and B-type flagellins [87]. reFliC-FN showed good biocompatibility and safety, highlighting ferritin’s potential as a delivery system and reFliC-FN as a promising PA vaccine candidate. These results confirm the broad applicability of self-assembling nanoparticles in vaccine development.

## 7. The Potential and Challenges of Self-Assembling Ferritin Nanoparticles in Vaccine Development

Self-assembling ferritin-based vaccines face limitations in development and clinical use, such as high production costs, complex engineering, and challenges with antigen stability and aggregation. Furthermore, regulatory hurdles and potential immunogenicity of ferritin may lead to adverse reactions.

### 7.1. Advantage and Disadvantage of Self-Assembled Ferritin in Developing Vaccines

Self-assembling ferritin nanoparticles offer a unique set of advantages over other nanomaterials in vaccine development. One significant advantage is their inherent biocompatibility and biodegradability, as ferritin is a naturally occurring protein in many organisms, which minimizes the risk of toxicity and adverse immune responses. Ferritin can self-assemble into highly uniform and stable nanocages, which provides consistent antigen presentation, a critical factor for inducing a strong and specific immune response. Moreover, ferritin’s structure allows for the genetic or chemical modification to display multiple copies of antigens on its surface, thereby enhancing the immunogenicity of the vaccine. Another advantage is that ferritin nanoparticles can encapsulate and co-deliver antigens and adjuvants, potentially boosting the immune response by providing a synergistic effect [76,77]. However, ferritin nanoparticles also have some limitations. One challenge is that the fusion of antigens to ferritin might alter the conformation of the antigens, potentially reducing their immunogenicity or leading to improper folding. Additionally, solubility and stability issues may arise with engineered ferritin–antigen complexes, potentially resulting in aggregation or loss of function. Another potential drawback is the immunogenicity of ferritin itself; repeated exposure might lead to the development of an immune response against the ferritin protein, which could limit its use in certain vaccine applications, particularly those requiring multiple doses. Despite these challenges, the advantages of ferritin, such as ease of production and ability to induce strong immune responses, make it a promising delivery system [93]. It will provide an excellent delivery platform for novel vaccines and targeted therapeutics, such as mRNA vaccines or CRISPR-Cas9-based therapies.

### 7.2. The Limitations and Challenges of Ferritin-Based Vaccines in Clinical Applications

In clinical applications, ferritin-based vaccines present several potential limitations and challenges. One significant challenge is the manufacturing cost. The production of engineered ferritin nanoparticles at a large scale is expensive due to the complexity of the protein engineering and purification processes. Additionally, ensuring the consistency and quality of the final product will further increase costs. Another challenge lies in the technical barriers associated with the engineering of ferritin vaccines. The fusion of antigens to ferritin must be carefully designed to preserve the antigen’s conformation and immunogenicity, while also maintaining the stability and solubility of the ferritin nanoparticles. Achieving this balance is technically demanding and may require extensive optimization. Regulatory challenges also pose a significant hurdle. As with any novel vaccine platform, ferritin-based vaccines must undergo rigorous testing to ensure safety and efficacy. The regulatory approval process for such vaccines can be lengthy and complex, requiring comprehensive preclinical and clinical trials to meet the stringent requirements set by regulatory agencies. This process can be further complicated by the need to address potential immunogenicity concerns related to the ferritin protein itself [9,94].

Overall, while ferritin-based vaccines hold great promise, these challenges highlight the need for continued research and development to overcome technical and regulatory barriers, as well as strategies to reduce manufacturing costs, to facilitate their successful clinical application.

## 8. Conclusions

Ferritin, as a paradigm of genetically engineered subunit recombinant vaccines, stands out for its excellent safety, stability, and biocompatibility. These intrinsic properties not only help mitigate potential side effects but also enable ferritin nanoparticle vaccines to stabilize antigenic units, allowing the presentation of multivalent antigens and enhancing the effectiveness of immune responses. Various methods, including genetic engineering modifications, can display multiple peptides or protein antigens on the ferritin surface. This ease of genetic modification provides technical support for the development of ferritin nanoparticle vaccines. Ferritin’s trimeric spatial structure and multiple antigen presentation capabilities distinguish it from other vaccine carriers, making it a highly promising platform for vaccine delivery. Ferritin nanoparticles have been widely used for the delivery of drugs and viral and bacterial antigen proteins. The unique properties of nanoparticle-anchored antigens helped optimize subunit vaccination strategies, providing advantages in cellular uptake, antigen presentation, and overall immune response. As scientific exploration progresses, the unique attributes of ferritin will gradually be unveiled, injecting new impetus into the development of ferritin-based vaccines.

## Figures and Tables

**Table 1:** Recent advance of the development of ferritin nanoparticles vaccines.

Antigens	Target Pathogens	Attached Region of Ferritin	Expression System	Experiment Animal	Immunological Effects of the Ferritin Nanoparticles Vaccine	References
E protein of ZIKV	Zika virus (ZIKV)	N-terminus	*E. coli*	Mice	Enhanced high-affinity antigen-specific IgG antibody levels, increased secretion of the cytokines IL-4 and IFN-γ by splenocytes, significantly activated T/B lymphocytes, and improved the generation of memory T/B cells.	[78]
E2 protein	Classical swine fever virus (CSFV)	N-terminus	SF9 cells	Rabbits	Elicited higher neutralizing antibody titers and significantly induced CSFV-specific IFN-γ-secreting cells compared to monomeric E2 protein.	[52]
BA.5 RBD	Severe acute respiratory syndrome coronavirus-2 (SARS-CoV-2)	N-terminus	Through Fc-Protein-A-tag-mediated conjugation	Golden hamsters	Stimulated strong innate and adaptive immune responses, inducing cross-neutralizing antibodies and protecting golden hamsters from multiple SARS-CoV-2 variants	[45]
Conserved stem domain of hemagglutinin, ectodomain of matrix protein 2	Influenza Virus	N-terminus	*E. coli*	Mice	Induces cross-reactive neutralizing antibodies, cellular immunity, and long-lasting memory B cell responses, offering protection against H3N2 and partial protection against H1N1.	[79]
Three copies of extracellular domain of the transmembrane protein M2 of H1N1	Influenza Virus	N-terminus	*E. coli*	Mice	Significantly activated lung CD11b dendritic cells, increased effector memory T cells and tissue-resident memory T cells in the lungs, and boosted mucosal IgG and IgA antibody production.	[57]
Dominant B and T cell epitopes of the highly immunogenic ASFV antigen (p72, CD2v, pB602L and p30)	African swine fever virus (ASFV)	N-terminus	*E. coli*	Mice	The nanoparticle vaccines can induce a more robust T cell response, and the high-level antibody response against ASFV can last for more than 231 days.	[75]
E protein domains I and II (EDI–II) of DTMUV (EDI–II-RFNp)	Duck Tembusu virus (DTMUV)	N-terminus	*E. coli*	Ducks	The self-assembled ferritin nanoparticles effectively protect ducks against the DTMUV challenge.	[80]
Hemagglutinin protein	Peste des Petits Ruminants (PPR)	N-terminus	Silkworm baculovirus	Mice	The immunogenicity and protective immune response of H-Fe nanoparticle antigens expressed by silkworms were improved compared with the H antigen alone.	[81]
Ectodomain of BPIV3 hemagglutinin-neuraminidase (HN)	Bovine parainfluenza virus type 3(BPIV3)	N-terminus	Baculovirus	Mice	The nanoparticles induced dendritic cell maturation, upregulated surface molecules, increased inflammatory cytokine secretion, and enhanced T cell activation. In mice, it induced higher titers of specific antibodies and provided better protection against BPIV3.	[82]
Hemagglutinin	Canine distemper virus (CDV)	C-terminus	*E. coli*	Mice	All proteins self-assembled into nanoparticles. Vaccination induced strong, long-lasting antibody responses and anti-CDV neutralizing activity, particularly in YaH4F and YaHF groups, which also enhanced ADCC effects and induced Th1 and Th2 responses.	[83]
E2 protein	Hepatitis C virus (HCV)	N-terminus	Drosophila Schneider 2 cell	Mice	The sE2-ferritin nanoparticle not only had nearly natural conformation but also had better affinities than the unfused sE2 to neutralizing antibodies, receptor, and patient serum. Mouse immunization studies showed that sE2-ferritin was more potent than sE2 in inducing anti-HCV broadly neutralizing antibodies.	[84]
Inner capsid protein VP6	Rotavirus	N-terminus	*E. coli*	Mice	Oral administration in mice induced strong humoral and mucosal immunity. Transgenic expression in mouse milk also induced strong immunogenicity in pups, reducing diarrhea symptoms and growth impacts.	[85]
The trimeric *N. meningitidis* antigen, NadA.Two fragments of NadA(f NadA5 and NadA3)	Neisseria meningitidis	N-terminus	*E. coli*	Mice	In mice, the two nanoparticles elicited comparable NadA antibody levels that were 10- to 100-fold higher than those elicited by the corresponding NadA trimer subunits. Further, the NadA ferritin nanoparticles potently induced complement-mediated serum bactericidal activity.	[86]
Type A flagellin	*Pseudomonas aeruginosa* (PA)	N-terminus	*E. coli*	Mice	A-type flagellin- ferritin nanoparticles induced a strong Th1 immune response and enhancing protection against PA strains with A-type and B-type flagellins.	[45]
PcrV and OprI of PA	*Pseudomonas aeruginosa* (PA)	N-terminus	*E. coli*	Mice	Intramuscular immunization with nanoparticles induced quick and efficient immunity and conferred protection against PA pneumonia in mice. In addition, intranasal immunization with nanoparticles enhanced protective mucosal immunity.	[87]
VP1	Foot-and-mouth disease (FMD)	N-terminus	*E. coli*	Mice, pig	The results from guinea pigs immunized with Hpf-T34E showed that the immune efficacy was largely consistent with the immunogenicity of the FMD inactivated vaccine (IV) and could confer partial protection against FMDV challenge in guinea pigs.	[42]
GP5 Protein	Porcine reproductive and respiratory syndrome virus (PRRSV)	N-terminus	*E. coli*	Mice	Ferritin (Ft) nanovaccines targeting the major glycoprotein (GP5GP5m-Ft t) exhibited the highest ELISA antibody levels, neutralizing antibody titers, the lymphocyte proliferation index, and IFN-γ levels. Furthermore, vaccination with the GP5m-Ft nanoparticle effectively protected piglets against a highly pathogenic PRRSV challenge.	[88]
PEDV HR protein	Porcine epidemic diarrhea virus (PEDV)	N-terminus	*E. coli*	Mice	HR-Ferritin nanoparticles stimulated the maturation DCs and elevated the secretion of pro-inflammatory cytokines, while enhancing the uptake of antigens by DCs.	[89]

## Data Availability

No new data were created or analyzed in this study.
